# Supplementary material for: The causal role of circulating inflammatory markers in osteoporosis: a bidirectional Mendelian randomized study
Source: Front Immunol. 2024 Jul 18;15:1412298. doi: 10.3389/fimmu.2024.1412298 (PMC11291241; doi:10.3389/fimmu.2024.1412298)
Supplement: Supplementary file 1 [file DataSheet_1.zip › supplementary material/OSTEOPOROSIS-CIM/GCST90274830.tsv.gz_leave_one_out.pdf]

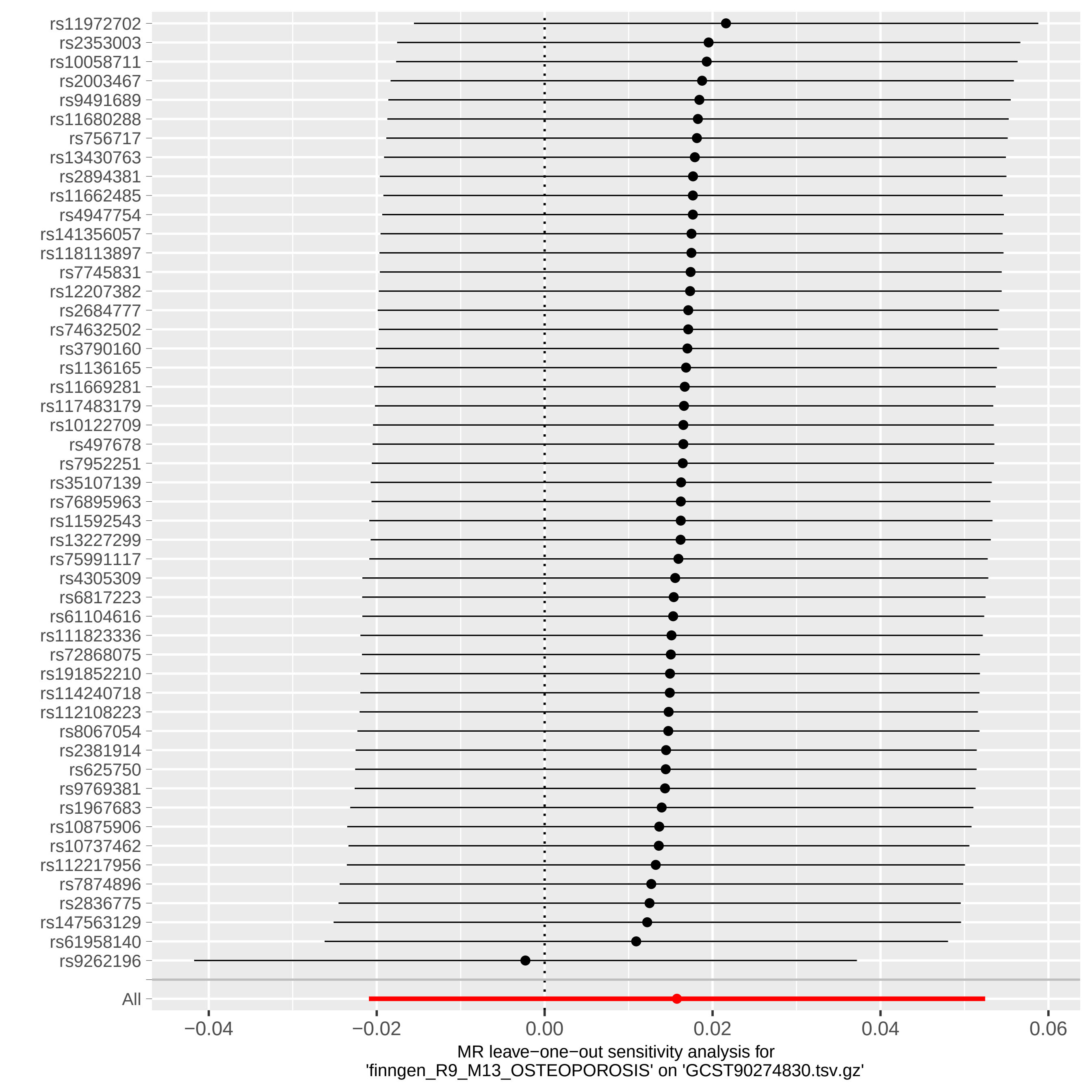

rs11972702
rs2353003
rs10058711
rs2003467
rs9491689
rs11680288
rs756717
rs13430763
rs2894381
rs11662485
rs4947754
rs141356057
rs118113897
rs7745831
rs12207382
rs2684777
rs74632502
rs3790160
rs1136165
rs11669281
rs117483179
rs10122709
rs497678
rs7952251
rs35107139
rs76895963
rs11592543
rs13227299
rs75991117
rs4305309
rs6817223
rs61104616
rs111823336
rs72868075
rs191852210
rs114240718
rs112108223
rs8067054
rs2381914
rs625750
rs9769381
rs1967683
rs10875906
rs10737462
rs112217956
rs7874896
rs2836775
rs147563129
rs61958140
rs9262196
All
−0.04
−0.02
0.00
0.02
0.04
0.06
MR leave−one−out sensitivity analysis for
'finngen_R9_M13_OSTEOPOROSIS' on 'GCST90274830.tsv.gz'
